# Thermal Degradation of Fractionated High and Low Molecular Weight Polyisobutylene

**DOI:** 10.6028/jres.068A.014

**Published:** 1964-04-01

**Authors:** D. McIntyre, J. H. O’Mara, S. Straus

## Abstract

A series of thermal degradation studies on polyisobutylene has been carried out at relatively low degradation temperatures using samples of high purity, both fractionated and unfractionated, and of both high and low molecular weight. Rate curves of the high molecular weight polymer show maximums while those of the low molecular weight polymer show large initial rates which steadily decrease with increased volatilization. Rates of degradation of all samples become similar with increased volatilization. Rate studies indicate strong random initiation with the initial rate of volatilization showing very little dependence on molecular weight. The drop in molecular weight with increased degradation and the lack of broad maximums at moderate values of conversion shows the influence of an appreciable amount of transfer and a low kinetic chain length. The rate of degradation is found to be much faster than that of polypropylene or polyethylene probably because polyisobutylene disproportionates and forms radicals more easily. Equations are suggested for these degradation reactions.

## 1. Introduction

The thermal degradation of polymers has been extensively studied in recent years. Numerous theoretical and experimental studies have shown that the thermal degradation of some polymers can be described by depolymerization mechanisms involving only random or end initiation. For example, linear polyethylene appears to have a purely random initiation of carbon-carbon bond breaks along the chain backbone, whereas polytetraffuoroethylene has only end-initiated carbon-carbon chain breaks. Different mechanisms for such widely different chemical structures can be explained on the basis of the known differences in the carbon-carbon bond strengths and the relative reactivities of hydrogen and fluorine covalent bonds. However, the degradation experiments and their interpretation become more complicated when the substituents on the carbon-carbon chain exert more subtle chemical effects. In these cases chain transfer reactions must be considered as possible reaction paths, in addition to combined end and random initiation. A very laborious and systematic experimental study may be necessary to sort out their relative contributions to the degradation.

The systematic study of the polyolefin system allows an interesting comparison of the effects of chemical structure. Wall and Straus [1]^1^ have recently compared the different rates of thermal degradation of linear and branched polyethylene, linear and branched polypropylene, and polyisobutylene. Polyisobutylene shows a significant departure from the degradation results of the other linear polymers in the polyolefin series. It is much more unstable and does not exhibit a maximum in the rate of volatilization. This comparison was based upon earlier kinetic observations of polyisobutylene by Madorsky and Straus [2] over a 20-deg temperature interval and at moderate initial rates of volatilization.

In order to gain greater insight into the mechanism of the polyisobutylene degradation, it is necessary to evaluate initial rates of degradation over a large range of molecular weights, as well as the molecular weight changes during the degradation experiment. For this purpose a series of thermal degradation experiments was carried out at relatively low degradation temperatures using a balance recently utilized by Madorsky [3] for measuring low rates of volatilization, and with polyisobutylene samples of high purity, both fractionated and unfractionated, and of both high and low molecular weight.

## 2. Materials

### High molecular weight polyisobutylene

**Figure 1:**
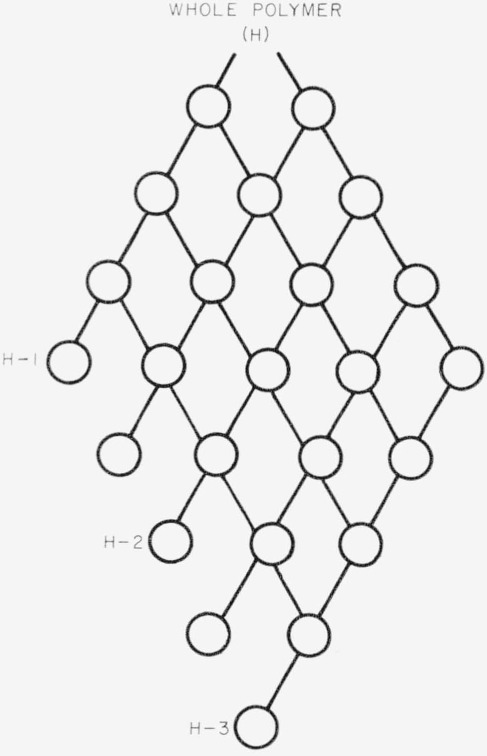
Triangular refractionation scheme.

The high molecular weight polyisobutylene was prepared by Esso Research Company by a BF_3_ initiated polymerization of isobutylene. Fractionation of the whole polymer was begun with a 0.4 percent solution in benzene and *n*-heptane (1:2). As the fractionation proceeded the solution was made progressively richer in benzene. A refractionation scheme was used as shown in figure 1. Distilled acetone was used as the precipitant. Six final fractions were obtained. Polymer samples H–1, H–2, and H–3 represent the first, fourth, and sixth fractions.

### Low molecular weight polyisobutylene

The low molecular weight whole polymer was a sample of commercially available whole polymer supplied by the Enjay Company of the Esso Standard Oil Company and designated as Vistanex LM, Type MS. Fractionation of the whole polymer was begun with a 1 percent solution in benzene using distilled acetone as a precipitant. Six fractions were obtained, with the first fraction representing about 50 percent of the whole polymer. The remaining fractions were about equal in size. Polymer sample L–3 was the third fraction of this fractionation.

The large first fraction was refractionated using a 1 percent solution in benzene with distilled acetone as the precipitant. Five fractions roughly equal in size were obtained. Polymer sample L–2 was the fourth fraction.

The first fraction of this second fractionation was again refractionated from a 1 percent solution in benzene into three fractions using distilled acetone as the precipitant. Polymer sample L–l was the first fraction of this fractionation.

Pertinent data on the fractions and whole polymers studied are given in table 1. The molecular weights of these materials were determined by the viscosity method using the relation [*η*] = 1.8×10^−4^ *M*^0.68^ [4]. The intrinsic viscosities were determined in iso-octane at 40 °C with an Ubbelohde viscometer.

## 3. Experimental Procedures and Results

### 3.1. Degradation Procedure

The thermal degradation studies were done using an electronic microbalance that automatically recorded the temperature and loss of weight of the sample and could be operated for long periods of time without attention. Measurements were made at 298.5 °C.

The experimental procedure using this balance has been described in detail [3]. A small platinum crucible containing 4 to 5 mg of polymer was suspended from the arm of the balance enclosed in a glass housing. The system was operated at a pressure of about 10^−5^ mm Hg. The sample was heated by placing a preheated electric furnace around the glass housing. The platinum crucible reached operating temperature in about 15 min, and it was found that very little weight loss occurred during this time. Temperatures were measured by using a chromel-constantan thermocouple directly under the crucible. This thermocouple was precalibrated against a similar thermocouple placed inside the crucible in contact with its bottom. The temperature was kept constant to within ±0.2° C by means of an electronic thermostat.

### 3.2. Rates of Degradation

**Figure 2:**
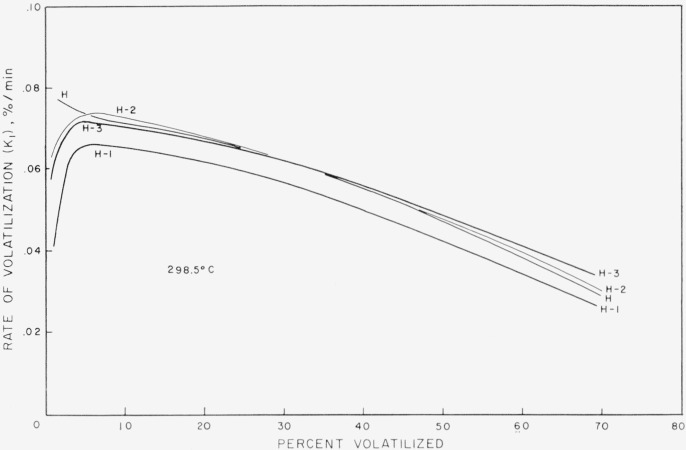
Percent of sample volatilized per minute as a function of percentage volatilized for high molecular weight polyisobutylene.

**Figure 3:**
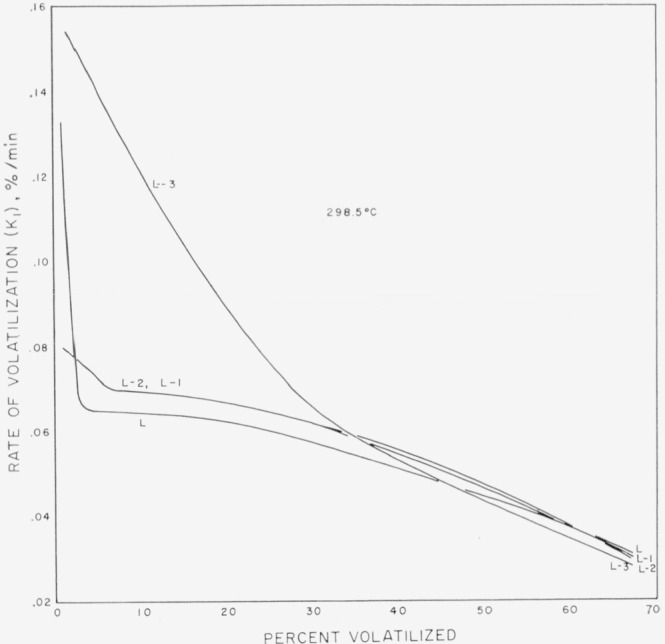
Percent of sample volatilized per minute as a function of percentage volatilized for low molecular weight polyisobutylene.

The extents of degradation of the polyisobutylene samples are given in table 1. The rates are plotted in figures 2 and 3 in percent of initial sample volatilized per minute, *K*_1_, as a function of percent volatilized.

The high molecular weight fractions all show, after the establishment of the predetermined temperature, a rapid rise of volatilization in rate to a maximum rate of about 0.06 to 0.07 percent volatilization per minute at about only 4 percent loss (fig. 2). Then the rates decrease slowly to about 0.04 percent volatilization per minute at about 70 percent loss. The whole polymer shows an initial high rate of volatilization which decreases rapidly so that, after about 5 percent loss, the rate of volatilization follows that of the fractions.

The low molecular weight whole polymer shows a very rapid initial degradation rate, over 0.13 percent volatilization per minute, which drops very rapidly to about 0.065 percent loss per minute at only about 4 percent loss of sample (fig. 3). The rate then levels off, decreasing somewhat more rapidly as volatilization proceeds. The two higher fractions of the low molecular weight polymer also show an initial rapid rate of volatilization of much less magnitude than that of the whole polymer. After the initial 5 percent loss, these two fractions follow essentially the same rate curve as the whole polymer.

The lowest fraction of the low molecular weight polyisobutylene shows the highest initial rate, over 0.15 percent volatilization per minute. This fraction continues to show a high rate of volatilization which decreases slowly up to about 31 percent loss of sample. At this point the rate becomes essentially that of the other fractions and whole polymer.

### 3.3 Molecular Weight of Degraded Polymers

Five samples of polyisobutylene fraction H–2 were pyrolized in a vacuum for various periods of time at a temperature of 300 °C.

**Figure 4:**
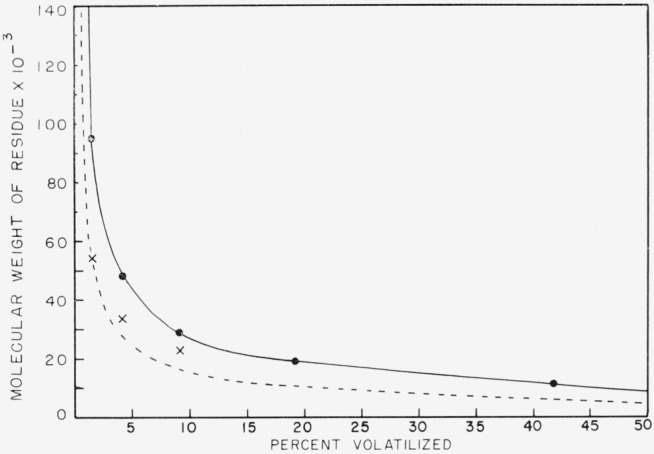
Drop in the molecular weight of polyisobutylene, fraction H–2, molecular weight 1,980,000. ●, viscosity measurements; x, osmotic pressure measurements;….,represents calculated curve for a constant ratio of *M_v_/M_n_* based on the results from the 1.5 percent volatilized sample.

After pyrolysis, the molecular weights of the residues were determined by viscosity and osmotic pressure methods. The osmotic pressure determinations were made in benezene at 22 °C in a Stabin osmometer fitted with a “never-dried” gel-cellophane No. 600 membrane. All of the measurements were equilibrium osmotic pressures and required about eight days to come to equilibrium for the lower molecular weight samples. Results of these pyrolysis experiments and molecular weight determinations are shown in table 2. In figure 4 the molecular weight of the residue is plotted against the percent volatilized.

Meaningful osmotic pressure measurements could not be made on the last two samples because of solute leakage through the membrane resulting in unstable, drifting osmotic pressures. Small leakage of the smaller molecules in the other samples most probably also accounts for the apparent narrowing of the distribution as given in the last column of table 2. The dotted line is drawn by assuming that the ratio of viscosity average to number-average molecular weight is constant after 1.5 percent volatilization.

The molecular weight measurements indicate a very rapid drop in molecular weight from 1,980,000 to 95,000 with just 1.5 percent loss of weight. The molecular weight drops all the way to 28,000 during the initial 10 percent loss in weight, but then drops less drastically as volatilization proceeds.

## 4. Discussion

The results of the degradation rate and molecular weight studies very strikingly demonstrate the absence of end initiation and the presence of predominantly random initiation. The rate studies very clearly indicate that over a molecular weight range of 50-fold the initial rate of the volatilization shows very little dependence on molecular weight. There may be some question about the value of the initial rate for the high molecular weight H series, but within the experimental accuracy of the data the curves may be considered to be equivalent. The existence of the maximum in the curves is beyond experimental error and must be explained. The upward curvature for the whole polymer is unquestionably related to the existence of a very broad molecular weight distribution in the whole polymer, the low molecular weight portion of which quickly distills out. Even with the most diligent search for a molecular weight effect in the initial rate, one can only find a doubling of the rate for a molecular weight change of 50 times. What is even more significant is the fact that the extrapolation of the rate curves after only 4 percent volatilization yields values of 0.07 percent/min and deviations no greater than 0.005 from this value.

The drastic upward curvature of the rate of the very low molecular weight fraction (L–3) in figure 3 may indicate a large amount of low molecular weight constituent. When such broadly distributed polymers are fractionated it is extremely difficult to get very narrowly distributed fractions. However the fraction L–3 seems to indicate more low molecular weight material than might be expected from the fractionation. The degradation curve of the fraction does not become coincident with the whole polymer and the other fractions until volatilization reaches 30 percent. This result may indicate a definite change in degradation behavior at very low molecular weight.

The rate data reported here are qualitatively in agreement with the data reported by Madorsky and Straus [2]. However, the rates are about one-half those previously reported after temperature corrections are made. Inoue, Ouchi, and Yasuhira [5] also measured the rates of degradation and reported an activation energy of 41 kcal per mole in contrast to Madorsky’s value of 52 kcal per mole. The differences in rates may be due to small differences in the commercial preparation of the polymers. Thus, the chemical analysis of the volatiles from different samples gave 78 percent monomer in one case [6], and 20 percent in another [7]. Another less detailed study [8] indicates even less volatile matter. The preparative details of each of the reported polymers most likely differ from one another. The existence of the maximum in the volatilization rates has not been observed before, although the measurements by Madorsky were not made at such low rates or with as sharply fractionated materials. This maximum may simply be the result of the necessary buildup, in a random degradation, of components that can be distilled out.

The molecular weight study shown in figure 4 also shows the existence of a strong random initiation. The degree of polymerization changes to about 0.03 of the original within 1.5 percent degradation. This is to be compared with polyethylene studies which show a change to 0.002 of the original at the same conversion [9]. This drop in DP for polyisobutylene is not as large as might be expected for random initiation only and indicates the influence of an appreciable amount of transfer. Figure 4 does not extrapolate to low molecular weight volatiles at 100 percent conversion unless a very flat curve is drawn beyond 9 percent volatilization.

**Figure 5:**
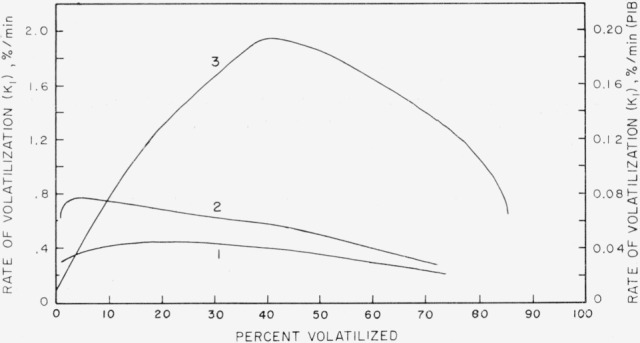
Comparison of the rates of volatilization for linear polymers. (1) Polyethylene at 400 °C [1]. (2) Polyisobutylene at 298.5 °C (ordinate at right). (3) Polypropylene at 375 °C [1].

Figure 5 shows a plot of different degradation rates for linear polyethylene, polypropylene, and polyisobutylene. The ethylene and propylene data are taken from Wall and Straus [1]. By assuming the value of 52 kcal per mole for the polyisobutylene thermal degradation and the rate at 298.5 °C, the polyisobutylene rate is calculated to be about 15 times that of the polypropylene at its maximum at 375 °C and 600 times faster than polyethylene [9]. This increased rate of thermal degradation is probably due to the easy disproportionation and radical formation in polyisobutylene. The lack of the broad maximum at a moderate value of conversion for the polyisobutylene must be explained on the assumption of a very small zip length and prevailing intermolecular and intramolecular transfer soon after the initial random break. This interpretation is in line with the molecular weight studies, and the analysis of volatiles. That only 20 percent of the monomer is distilled off lends support to the transfer reactions.

The reaction for the thermal degradation of polyisobutylene probably consists of thermal breaks of the bonds as shown in eq (1), followed in eq (2) by some rearrangements (intramolecular transfer) of the

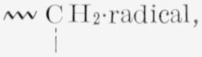
and disproportionation in eq (3) of the

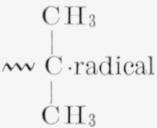
into monomer. Any of the radicals would be able to transfer hydrogen intemolecularly.

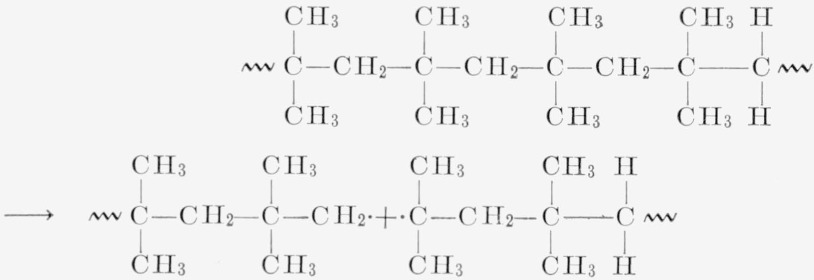
(1)

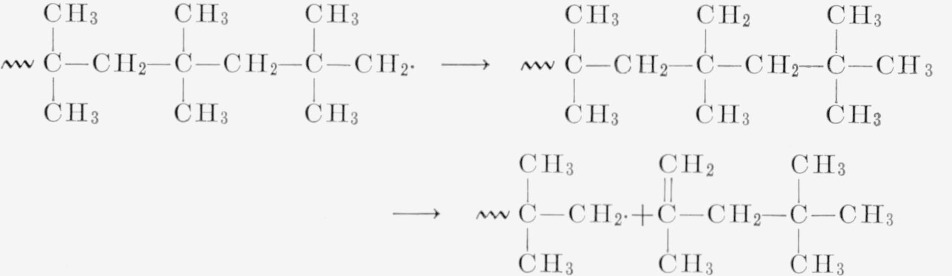
(2)

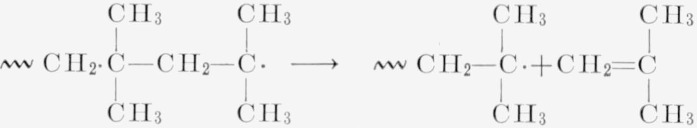
(3)

## Figures and Tables

**Table 1:** Degradation of polyisobutylene at 298.5 °C

Sample	Fraction	Mol. wt (*M_v_*)	Duration	Volatilization
				
			*hr*	%
H	Whole polymer	1,800, 000	23	71.3
H–1	High fraction	4,800, 000	25	83.8
H–2	Middle fraction	1,980, 000	23	71.6
H–3	Low fraction	700, 000	23	71.8
L	Whole polymer	49,000	23	68.3
L–1	High fraction	234, 000	23	70.3
L–2	Middle fraction	44, 200	23	69.2
L–3	Low fraction	24, 500	23	71.6

**Table 2:** Molecular weights of degraded polyisobutylene fraction H–2

Temperature	Duration of pyrolysis	Volatilization	*M_v_*(visc)	*M_n_*(osmotic)	*M_v_/M_n_*
					
*°C*	*min*	%			
300	24	1.5	95,000	54,800	1.73
300	70	4.2	48,100	33, 800	1.42
300	135	9.1	29,000	22, 900	1.27
300	260	19.2	19, 000		
300	660	42.0	11,400		

## Footnotes

1 Figures in brackets indicate the literature references at the end of this paper.
